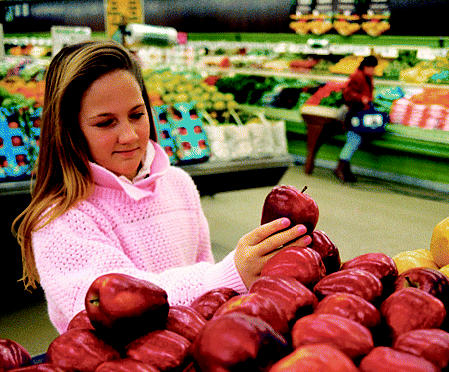# The Beat

**Published:** 2005-05

**Authors:** Erin E. Dooley

## The New Face of Herbs

After a meteoric rise in sales in the 1990s, use of botanical dietary supplements appears to have plateaued, according to a study in the 14 February 2005 issue of *Archives of Internal Medicine*. Still, exposure may continue to increase as more multivitamins now include botanical ingredients such as the carotenoid antioxidants lutein and lycopene (which may protect against macular degeneration and cancer). The study authors, from Boston University, wrote that the latter finding suggests that herbal supplements are gaining wider acceptance as evidenced by their incorporation into mainstream medicine. Furthermore, multivitamins are now being marketed not just as a source of daily allowances of vitamins and minerals but also as a means for preventing chronic diseases.

## Sunny Spanish Energy

Spain, known for its sunny climate, is turning that resource into energy. The Spanish Industry Minister has announced that as of 2005 any new or renovated buildings must be outfitted with solar panels. Spain’s government is seeking a 10-fold increase in the area of solar panels in use in Spain by 2010, and hopes to make the nation a leader in the use of renewable energy. The government has promised subsidies to help get the program off the ground, with details yet to be announced.

In making the announcement, the government stated that this measure could reduce greenhouse gases and save homeowners more than US$100 each year on fuel. According to additional government estimates, a single two-meter solar panel can cut a household’s yearly hot water bill by up to 70%.

## A Fresh Wind Blows in Beijing

Everyone knows China is a big place with a big population. Now it can add having the world’s largest wind power project to its list of superlatives. The new power plant, located 60 miles outside Beijing, will generate 400 megawatts per day, enough to power 240,000–400,000 households. This nearly doubles the amount of wind-generated power that China currently has. China announced in 2004 that it hopes to generate 12% of its energy from renewable sources such as wind by 2020.

The plant was planned as a means to help relieve Beijing from some of the world’s worst air pollution. Some 70% of the country is affected by acid rain formed in part by emissions from the coal-burning power plants that China currently relies on. The country is also fraught with power outages as demand outstrips supply.

## Butting Out of Bhutan

Known for its fierce protectiveness of its environment and culture, the Himalayan kingdom of Bhutan now has a unique place among the world’s nations—it is the first to impose a nationwide ban on public smoking and the sale of tobacco products. According to an act passed in July 2004 by the Royal National Assembly, selling tobacco products will result in a fine of US$225, a huge sum in this modest nation, and businesses caught in the act will lose their business licenses. Bhutanese bringing tobacco into the country from elsewhere will be charged a 100% tax and may smoke their tobacco only at home. Currently about 1% of Bhutanese are believed to smoke.

## Federal Agencies Pledge Computer Stewardship

The U.S. government represents 7% of the world demand for computers and is expected to spend $60 billion this fiscal year on information technology needs. Now the White House and 11 federal departments and agencies have signed a memorandum of understanding to develop and promote common strategies for more sustainable management of government computer resources. The signatories will put their purchasing power toward increasing demand for more energy-efficient and environmentally sustainable equipment; promoting implementation of optimal life cycle management practices for electronic equipment; reducing the economic and environmental costs of federal electronic equipment; and promoting the market and infrastructure for the reuse, demanufacturing, and recycling of obsolete equipment.

## Think Globally, Shop Locally

How much does that apple or carton of eggs really cost? A team of researchers from Britain’s University of Essex and City University tallied up the unaccounted environmental and transportation costs involved in bringing organic and conventionally grown produce to UK markets and published their calculations in volume 30, issue 1 (2005) of *Food Policy*. If Britons bought more organic produce and made their grocery trips by a means of transportation other than a car, the country would save more than US$7.5 million in impacts upon the environment. If all UK farms went organic, environmental costs would fall from almost US$3 billion to just over US$750 million. And if food came from within 12 miles of where it was consumed, environmental and congestion costs related to the transportation of food would fall from US$4.4 billion to US$440 million. Said coauthor Jules Petty, “The most political act we do on a daily basis is to eat, as our actions affect farms, landscapes, and food businesses.”

## Figures and Tables

**Figure:**
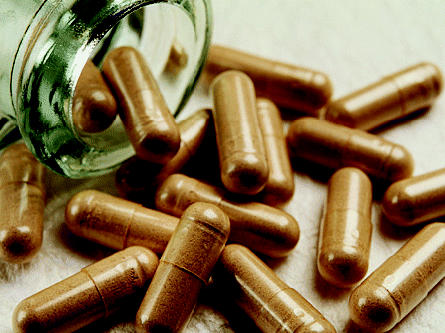


**Figure:**
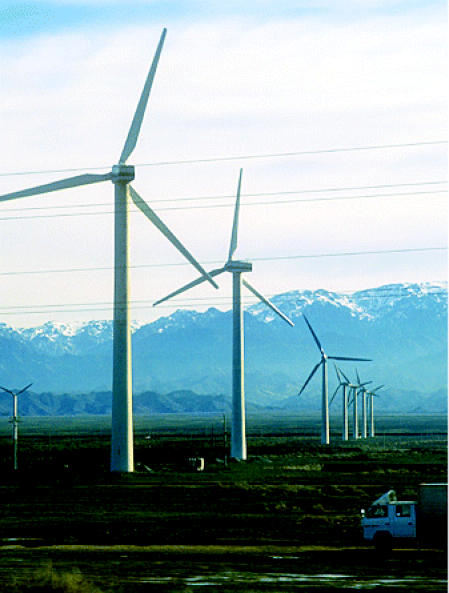


**Figure:**
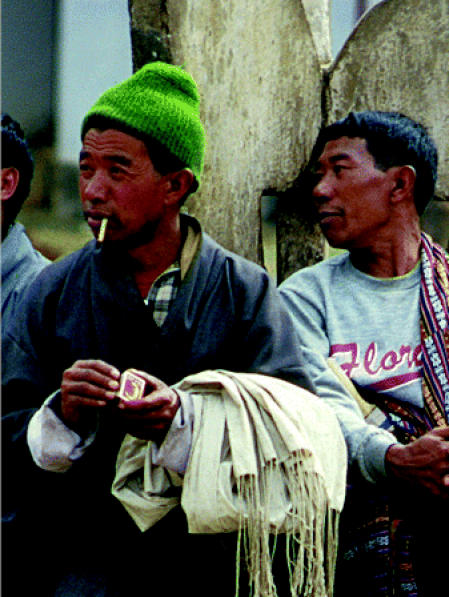


**Figure:**